# New Frontiers: ARID3a in SLE

**DOI:** 10.3390/cells8101136

**Published:** 2019-09-24

**Authors:** Joshua Garton, M. David Barron, Michelle L. Ratliff, Carol F. Webb

**Affiliations:** 1 Department of Chemistry and Biochemistry, University of Oklahoma, Norman, OK 73072, USA; joshgarton@ou.edu; 2 Department of Microbiology and Immunology, University of Oklahoma Health Sciences Center, Oklahoma City, OK 73104, USA; matthew-barron@ouhsc.edu; 3 Department of Medicine, University of Oklahoma Health Sciences Center, Oklahoma City, OK 73104, USA; michelle-ratliff@ouhsc.edu; 4 Departments of Medicine, Microbiology and Immunology, Cell Biology, University of Oklahoma Health Sciences Center, Oklahoma City, OK 73104, USA

**Keywords:** systemic lupus erythematosus, ARID3a, B lymphocytes, low-density neutrophils, plasmacytoid dendritic cells, interferon alpha

## Abstract

Systemic lupus erythematosus (SLE) is a devastating and heterogeneous autoimmune disease that affects multiple organs, and for which the underlying causes are unknown. The majority of SLE patients produce autoantibodies, have increased levels of type-I inflammatory cytokines, and can develop glomerulonephritis. Recent studies indicate an unexpected but strong association between increased disease activity in SLE patients and the expression of the DNA-binding protein ARID3a (A + T rich interaction domain protein 3a) in a number of peripheral blood cell types. ARID3a expression was first associated with autoantibody production in B cells; however, more recent findings also indicate associations with expression of the inflammatory cytokine interferon alpha in SLE plasmacytoid dendritic cells and low-density neutrophils. In addition, ARID3a is expressed in hematopoietic stem cells and some adult kidney progenitor cells. SLE cells expressing enhanced ARID3a levels show differential gene expression patterns compared with homologous healthy control cells, identifying new pathways potentially regulated by ARID3a. The associations of ARID3a expression with increased disease severity in SLE, suggest that it, or its downstream targets, may provide new therapeutic targets for SLE.

## 1. Introduction

The autoimmune disease systemic lupus erythematosus (SLE) affects approximately one million Americans [1], with symptoms ranging from rash and fatigue, to severe organ dysfunction [2,3]. Disease severity varies according to the degree of organ involvement, and the level of inflammation and systemic deposition of autoantibody-containing immune complexes, and is quantified using scores that combine measurements of these criteria as SLE disease activity indices (SLEDAI scores) [2,3]. The underlying mechanisms that lead to SLE are unknown, but several key features of the disease are present in the majority of patients. These include breaks in humoral tolerance that result in the production of autoimmune antibodies; inflammation typically characterized by increased levels of Type I cytokines, such as interferon alpha (IFNα); and, lupus-induced kidney damage, known as lupus nephritis. Renal involvement occurs in nearly 60% of SLE patients [4], and results in high healthcare costs [1] and mortality [5]. Very few effective therapies for SLE exist. Current treatments include Rituximab, an antibody that targets the majority of B lymphocytes, and Belimumab, an antibody that also targets B cells through the B cell survival factor, BLyS (B Lymphocyte Stimulator) or BAFF (B cell Activating Factor) [6]. Both drugs have broadly immunosuppressive effects through general B-cell inhibition, and fail to target the specific sources of autoimmune antibodies directly. Therefore, new therapeutic targets are badly needed. Our data now suggest that the DNA-binding protein ARID3a (A + T rich interaction domain protein 3a) is overexpressed in a number of hematopoietic cell types in SLE peripheral blood compared with healthy controls, and that expression of ARID3a is associated with increased SLEDAI scores. Although a number of contributing factors are likely to influence SLE pathology, in this review, we will highlight the molecular and cellular associations of ARID3a with the common lupus pathologies of autoantibody production, interferon induction, and the promotion of clinical nephritis.

**Figure 1:**
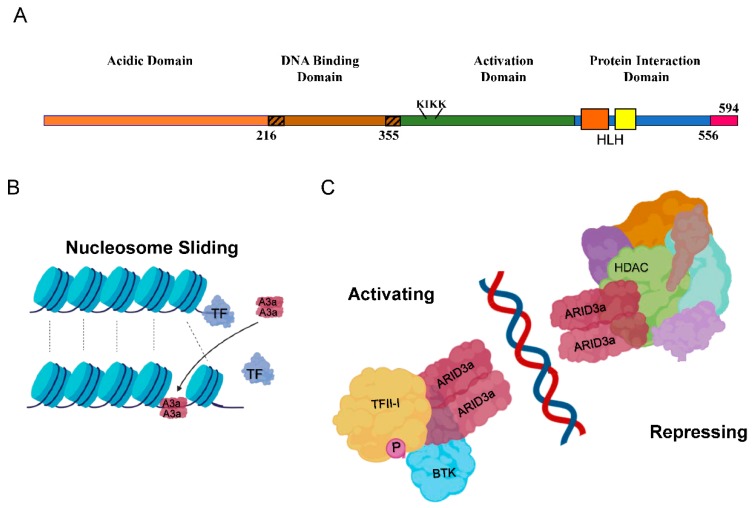
Schematic diagrams of ARID3a (A + T rich interaction domain protein 3a) domains and functions. (**A**) The four protein domains of ARID3a are shown, including the extended DNA-binding domain (hashed ends), the nuclear localization motif (KIKK), the helix-loop-helix regions (orange and yellow boxes), and amino acid numbers. The short carboxyl terminus has not been given a name or function. (**B**). ARID3a is proposed to induce nucleosome sliding, potentially disrupting the binding of other transcription factors, and potentially recruiting new transcription factors. (**C**). ARID3a DNA-interacting protein complexes can have either activating or repressing functions. Some of the known interacting proteins are shown. A3a—ARID3a; TF—transcription factor; HDAC—histone deacetylase; TFII-I—transcription factor II-I; P—phosphorylation; BTK—Bruton’s tyrosine kinase.

ARID3a, or Bright (B cell regulator of immunoglobulin (Ig) heavy chain transcription) as it was first called in the mouse, was originally discovered as a transcription factor that increases Ig-heavy chain transcription and binds to the intronic heavy chain enhancer, where it is associated with epigenetic effects and the organization of chromatin into transcriptionally active domains by interactions with nuclear matrix associated regions [7,8,9,10,11]. ARID3a is not required for the initiation of Ig production, but requires dimerization and association with both Bruton’s tyrosine kinase (BTK) and TFII-I (Transcription Factor II-I) for activity in B lymphocytes [8,12,13]. Binding sites for ARID3a were first identified 5′ of the promoter of the V1 S107 gene, a gene necessary for responses to phosphorylcholine [14]. In humans, there are 15 ARID family proteins, most of which have epigenetic functions, and are members of larger chromatin-modifying complexes that act in part through protein domains associated with discrete epigenetic functions [15,16,17]. The three members of the ARID3 family are distinguished as smaller proteins that have an extended DNA-binding domain that confers increased sequence specificity compared to other ARID family members, but lack obvious protein domains associated with epigenetic functions (Figure 1A). Because ARID3a can act as both an activator and suppressor of transcription [8,9,18], ARID3a may regulate gene expression as a DNA-specific tether that recruits other epigenetic modifiers to those sites (Figure 1B,C).

**Figure 2:**
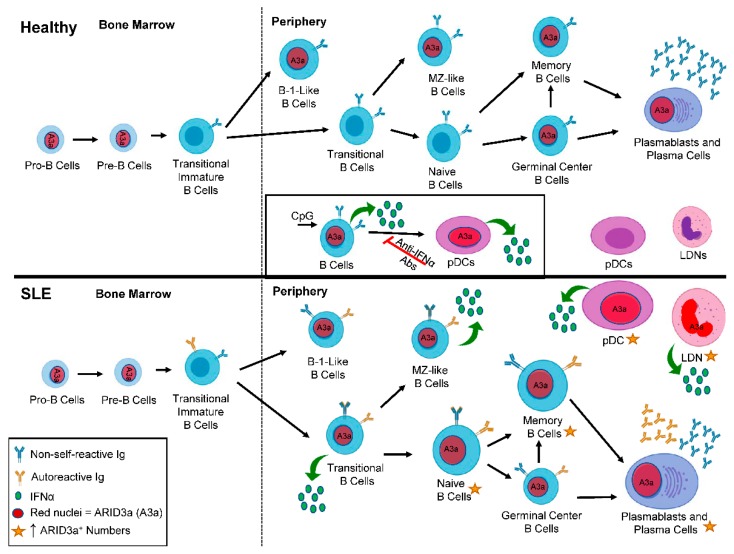
ARID3a expression is enhanced in systemic lupus erythematosus (SLE) B cell subsets compared to healthy controls. A diagram depicts B lineage development in healthy (top panel) and SLE patients (bottom panel) from the bone marrow to the periphery. Red nuclei denote cell subsets that express ARID3a. The inset shows the induction of ARID3a and interferon alpha (IFNα; green circles), and the effect of those ARID3a-expressing B cells on healthy pDCs. In SLE patients, larger cells and gold stars indicate those cell subsets with increased numbers of ARID3a^+^ cells and associated IFNα production. Blue immunoglobulin (Ig) indicates non-self-reactive Ig, while gold Ig indicates the subsets of cells that can also produce autoreactive Ig. pDCs—plasmacytoid dendritic cells; LDN—low density neutrophils; A3a—ARID3a.

Although most ARID family members are expressed ubiquitously, ARID3a expression is tissue and developmentally restricted to hematopoietic and other adult stem cells, and to particular types of mature cells in the hematopoietic lineage [19]. The expression of ARID3a is tightly regulated during B cell development in both mice and humans, and is the highest in the bone marrow pre-B and germinal center-activated B cells, while other B cell subsets, including the majority of mature splenic B cells, lack both ARID3a mRNA and protein [20,21]. During B lymphocyte development in mice, ARID3a expression is tightly regulated, such that it is expressed in transitional T1 B cells, down-regulated at the T2 cell stage where tolerance checkpoints have been identified [22,23], and turned off at the level of transcription in naïve follicular B cells [24,25]. Others have shown recently that ARID3a is required for fetal lineage B1 B cell development [21,26]. ARID3a is also expressed in mature B1 and marginal-zone (MZ) B cells, two cell types associated with autoimmunity in several systems [24,27,28,29,30,31]. Figure 2 summarizes where ARID3a is expressed during healthy B lymphocyte development.

## 2. ARID3a and Autoantibodies in SLE

Several years ago, we discovered that the constitutive expression of murine ARID3a/Bright in all B lymphocyte lineage cells in transgenic mice resulted in antinuclear antibody (ANA) production in young mice and immune complex deposits in the kidneys of older mice [27,28]. ANAs and anti-DNA antibodies typically increase during flares of SLE disease activity and are hallmarks of lupus in 50%–70% of SLE patients [27,32]. ANA production in ARID3a-transgenic mice was observed on two genetic backgrounds and was associated with increases in transitional T1 B cells and MZ B cells [28], subsets previously associated with autoimmune disease activity in mice and humans [31,33,34,35]. In addition, chimeric mice generated from hematopoietic stem cells taken from the transgenic mice also developed ANAs and increased MZ B cell subset numbers, indicating that the constitutive ARID3a expression was directly linked to the production of ANAs and increases in MZ cells [27].

To determine if ARID3a was over-expressed in lupus B lymphocytes, we assessed the numbers of peripheral blood B cells for ARID3a expression in healthy controls, patients with rheumatoid arthritis, and SLE patients [36]. Surprisingly, SLE circulating blood peripheral B lymphocytes had dramatically increased ARID3a expression [36]. Examination of a random cross section of 115 lupus patients revealed abnormally high numbers of circulating ARID3a^+^ B cells (up to 40-fold increases) compared with healthy controls and patients with rheumatoid arthritis [36]. ARID3a was expressed in all of the nine SLE B cell subsets we examined, including naïve B cells that typically do not express ARID3a transcripts in healthy controls (Figure 2) [20,36]. Longitudinal analyses of 37 SLE patients revealed that numbers of ARID3a-expressing B lymphocytes varied over time in each patient and within individual B cell subsets, but the total numbers of ARID3a-expressing B cells, irrespective of the subset, were associated with increased SLEDAI scores (*p* = 0.0039) [36]. ARID3a expression in each B cell subset was bimodal, with only a fraction expressing ARID3a, and there was no direct relationship with specific organ involvement or any autoantibody specificity [36,37]. ARID3a was expressed in both healthy and SLE MZ-like B cells, suggesting it may have innate immune functions in those cells [36,38]. Intriguingly, Epstein Barr virus (EBV) exposure has been associated with increased lupus susceptibility [39], and some anti-DNA antibodies cross react with the EBNA1 protein [39,40]. Others showed that EBV requires and recruits ARID3a for expression of the EBNA C promoter that maintains viral latency, associating this virus with ARID3a expression [41]. Thus, ARID3a likely plays important roles in innate immunity in healthy B cells, and may be over-expressed in SLE in a fashion similar to our ARID3a transgenic mice that developed autoantibodies.

**Figure 3:**
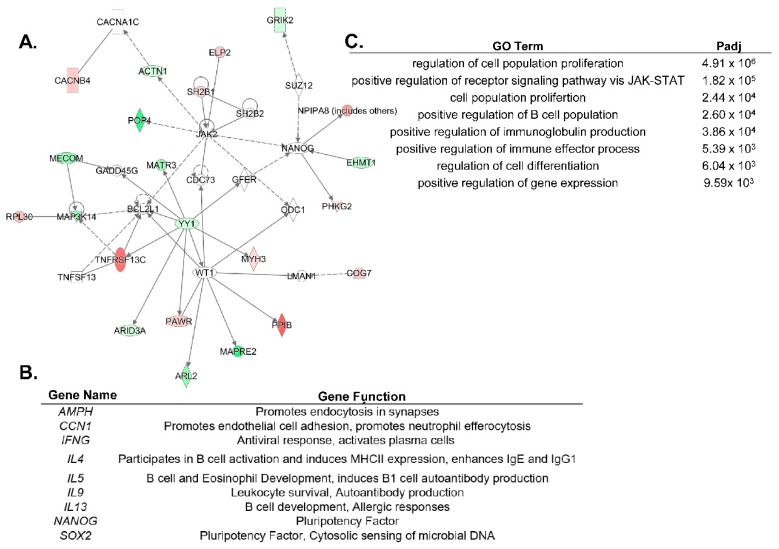
ARID3a expression is associated with diverse genes in distinct cell types. (**A**). An ingenuity pathway analysis (IPA) network was generated using genes differentially expressed in single cell RNAseq of naïve SLE B cells with a differential ARID3a expression. Green indicates upregulation and red indicates downregulation, while the genes associated with these pathways, but not differentially regulated in the B cells, are colorless. (**B**). SLE LDNs and pDCs shared nine differentially expressed genes associated with the ARID3a expression and listed here. (**C**). GO analyses of the nine ARID3a-associated genes differentially regulated in both pDCs and LDNs indicate the most significant pathways associated with their expression.

These data led us to hypothesize that ARID3a-expressing naïve B cells might be predisposed to produce autoantibodies. We sorted naïve B cells from both healthy controls and SLE patients, and generated 37 monoclonal antibodies from those cells, but failed to observe skewing toward specific Igs associated with autoimmunity in the SLE naïve B cells, perhaps because of the small number of Igs examined [37]. However, when we isolated total B cells from SLE patients with high versus low ARID3a-expression, and examined them for differential gene expression [38,42], we found associations with known mediators of disease activity. Specifically, many genes associated with IFNα expression (*IRF3, IRF5, IFI44*, and *OAS1*), toll-like receptors (*TLR9* and *TLR7*), and anti-apoptotic genes (*BCL2* and *BCL2L1*) were upregulated in the samples with increased ARID3a expression [42]. Additionally, our unpublished scRNA-seq data from naïve SLE B cells also show an upregulation of *OAS1*, *IFI27*, and *IRF3*, and confirm that *BCL2L1* is linked to pathways associated with ARID3a (Figure 3A). YY1, an important suppressor and epigenetic regulator, binds to sites in the IgH enhancer that overlap the ARID3a binding sites, and was co-expressed with ARID3a in naïve B cells from SLE patients (Figure 3A) [43]. Transcripts from 13 IFN signature genes were significantly upregulated in SLE B cells, and five of those, including *EPSTI1*, *IFI27*, and *OAS1*, were increased over 20-fold [42]. The gene signatures for the ARID3a-expressing B cells indicate a strong correlation to IFN, suggesting a potential connection between ARID3a and the lupus-associated cytokine, IFNα.

A number of labs demonstrated that SLE patients display altered epigenetic marks in peripheral blood cells [42,44]. Elegant studies by Scharer et al. indicate that SLE resting naïve B cells are already epigenetically distinct from healthy control B cells [44,45], suggesting that epigenetic changes might define unique transcriptomes in SLE. However, no epigenetic regulator was specifically associated with the signatures observed in these studies, and the data were obtained from all SLE patients, regardless of disease activities [45]. ARID3a has several characteristics of an epigenetic regulator. It alters chromatin accessibility of the Ig enhancer in mice ([10], our unpublished data), and the mouse homologue co-precipitated with at least one histone modifying enzyme [18]. In addition, our unpublished data suggest that ARID3a recruits epigenetic machinery. We found that whole blood from SLE patients with increased numbers of ARID3a-expressing B cells showed decreased methylation of promoters for a number of Type I interferon genes, including *IFNα2* and *IFNα6*, compared to SLE samples with normal levels of ARID3a-expressing B cells [38,42]. While these data do not directly associate ARID3a with open chromatin at those IFNα loci, these data suggested that ARID3a could be specifically associated with alterations in IFNα expression at the epigenetic level.

## 3. ARID3a and IFNα in SLE

Consistent with the data from other labs indicating that human B lymphocytes can secrete IFNα [46], the intracellular staining of SLE B lymphocytes revealed that IFNα is produced by a subset of B cells, most of which co-express ARID3a [38]. Indeed, we found that a subset of MZ-like healthy B cells can be stimulated with the TLR9 agonist CpG to increase ARID3a and IFNα expression [38]. EBV-transformed B cell lines express both ARID3a and IFNα, and were used to demonstrate that ARID3a inhibition reduced the production of both proteins [38]. These data, along with time course data indicating ARID3a expression could be induced in healthy cells prior to IFNα expression [38], link ARID3a with IFNα expression in B cells, and suggest that ARID3a may contribute to IFNα regulation.

The major sources of type-I IFN production in SLE are thought to be plasmacytoid dendritic cells (pDCs) and granulocytes, rather than B lymphocytes [47,48]. Therefore, we investigated if ARID3a-expressing B cells might have a helper cell activity and induce IFNα production in healthy pDCs. The addition of previously CpG-stimulated healthy donor B cells expressing ARID3a and IFNα to autologous pDCs upregulated IFNα production in the pDCs (Figure 2 inset) [38]. To our surprise, the pDCs that expressed IFNα also co-expressed ARID3a, emphasizing the association between ARID3a and IFNα expression, and demonstrating that mature cells other than B lymphocytes express ARID3a [38]. We next queried whether SLE patient pDCs and low-density neutrophils (LDNs) express ARID3a in association with IFNα. Indeed, we observed a very strong correlation between ARID3a and IFNα expression in lupus pDCs (Figure 2) [49]. However, the ARID3a and IFNα expression in the pDCs did not correlate highly with the SLEDAI scores, suggesting that other factors or cell types more strongly influenced disease activity [49].

LDNs are much more abundant than pDCs in SLE, comprising up to 54% of patient peripheral blood mononuclear cells, and they have been associated with both IFNα production and increases in autoimmune activity [50]. Neutrophils exposed to circulating chromatin produce significant amounts of IFNα in healthy individuals and in SLE patients [51]. We found that SLE LDNs also showed increased levels of ARID3a protein expression compared with LDNs from healthy controls (Figure 2) [49]. ARID3a was also strongly associated with IFNα expression in SLE LDNs, and was, surprisingly, more closely associated with increases in SLEDAI scores than IFNα levels [49]. These data support a role for ARID3a in IFNα-associated pathologies in SLE, and suggest that ARID3a may contribute to other disease-associated activities not directly correlated with IFNα levels. Indeed, others showed that IFNα levels do not associate well with SLE disease activity [52].

LDNs undergo spontaneous NETosis, a process whereby inflammatory extracellular nucleic acids decorated with histones, enzymes, and antimicrobial peptides capable of entrapping bacteria are extruded from cells, and this occurs at higher rates in LDNs than in normal density neutrophils [50,53,54]. This raises the possibility that LDNs may serve as a source for DNA and chromatin that could trigger TLR9 responses and reactivate self-reactive memory B cells [47,53,55,56,57]. Indeed, others showed that NETs that contact neighboring B cells can stimulate nucleotide-sensing TLRs and promote T-independent B cell activation [58]. In a lupus mouse model, the depletion of neutrophils reduced the splenic B cell numbers, serum BAFF, and anti-dsDNA IgG levels, and immune complex deposits in the kidneys [59]. In addition to NET production, LDNs and other neutrophils possess immunomodulatory properties capable of directly and indirectly stimulating autoimmune B cells [60,61,62,63]. B-helper neutrophil (N_BH_) subsets identified in the spleen by Puga et al. [58], produce the B-cell stimulating factors BAFF, APRIL (A Proliferation-Inducing Ligand), and IL-21, and parallel functions of T cells. N_BH_ cells also exhibit high rates of spontaneous NETosis, making them oddly similar to LDNs [58]; however, these cells have not been examined for ARID3a expression. Because ARID3a is over-expressed in lupus pDCs, LDNs, and B cells [36,38,49], we hypothesize that ARID3a contributes to interactions between these three cell types in lupus. Others found that bone marrow-derived neutrophils generate IFNα and stimulate early B cell precursors [64]. Therefore, inflammatory events in LDNs associated with ARID3a expression may precede the activation of autoimmune anti-self B cells in lupus. Indeed, recent studies indicate that transitional bone marrow B cells, the precursors of mature B cells in patients with SLE, already have an IFN and autoimmune signature [33].

Gene expression data from pDCs and LDNs with high levels of ARID3a protein provide new insights into genes associated with autoimmunity and increases in SLEDAI scores. Transcriptomic analyses of pDCs stratified by ARID3a protein expression levels identified 189 genes associated with both ARID3a and IFNα expression in pDCs, and only 122 differentially expressed genes with significant R^2^ values in LDNs [49]. These genes were tightly co-regulated in both cell types suggesting that ARID3a is either tightly associated with a master regulator of gene transcription, or that it is a master regulator itself [49]. Only nine differentially expressed genes were shared between LDNs and pDCs, in keeping with the cell-type specific effects of ARID3a on gene pathway regulation (Figure 3B,C) [49]. In addition, because ARID3a protein expression was more tightly associated with increases in the SLEDAI scores in LDNs than in pDCs, we postulate that the relatively small number of genes associated with ARID3a expression, but not with IFNα, will identify new mediators of disease activity in SLE. The ARID3a-associated LDN genes include eight transcription factors and two epigenetic factors that may function downstream of ARID3a, as well as a number of noncoding RNAs with potential regulatory functions.

## 4. ARID3a and Nephritis

While there is no direct evidence that ARID3a is associated with the development of nephritis in SLE patients, there are a number of interesting observations that suggest it could be. *NANOG* and *SOX2* are two genes frequently associated with ARID3a expression (Figure 3B), and with cell fate commitment [65,66,67]. These two genes play important functions in stem cells, and along with *OCT4*, may be regulated by ARID3a [18,68]. In *X. laevis*, ARID3a is expressed in kidney stem cells, where it binds to enhancers of the genes required for the regeneration of nephric tubules and changes histone 3 lysine 9 (H3K9me3) levels, allowing the expression of the *LHX1* gene critical for tubule formation [65]. Our unpublished data also reveal ARID3a expression in human adult kidney progenitor cells, suggesting it may play a role in human nephrogenesis as well. Additionally, resident renal cells secrete IFNα in a lupus nephritis mouse model [69], but it is not known whether IFNα secretion in these cells is associated with ARID3a expression. One might envision that the over-expression of ARID3a within kidney cells could alter gene expression patterns, contributing to inflammation and the autoimmune complexes observed in SLE that ultimately result in renal dysfunction.

## 5. ARID3a and Hematopoiesis

**Figure 4:**
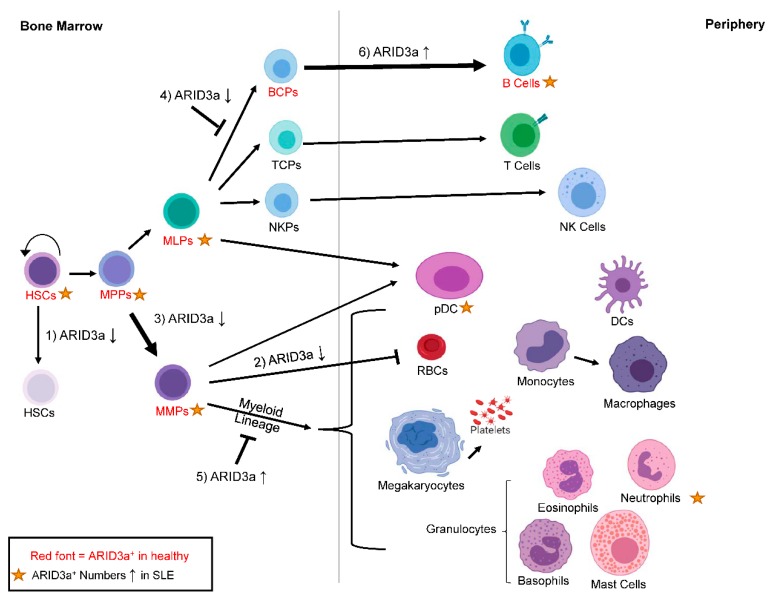
ARID3a is expressed in healthy and SLE hematopoietic progenitors, and the levels of ARID3a affect lineage decisions. Cell subsets with ARID3a expression in healthy individuals are indicated with the red font, while gold stars indicate populations with increased numbers of ARID3a-expressing cells in SLE patients. The effects of increased or inhibited levels of ARID3a (arrows up or down) on HSCs (**1**), RBCs (**2**) early myeloid lineage development (**3**), early B lymphoid development (**4**), late myeloid lineage development (**5**), and late B lineage development (**6**) are shown by thicker arrows for increases, or with a symbol showing that development is blocked. HSCs—hematopoietic stem cells; MPPs—multipotent progenitors; MMPs—multi-myeloid progenitors; MLPs—multi-lymphoid progenitors; TCPs—T cell progenitors; NKPs—natural killer cell progenitors; pDCs—plasmacytoid dendritic cells; RBCs—red blood cells.

ARID3a is also expressed in a number of hematopoietic progenitors [19,70,71], and is required for B lineage development in both mouse and man [70,72]. The knockdown of ARID3a in human cord blood leads to increases in myeloid lineage development, with associated reductions in the B lymphoid lineage [70]. Although the precise mechanisms of ARID3a function in stem cells have not been fully elucidated, knockout mice die between days 12 and 14 of gestation when hematopoiesis moves from the yolk sac to the fetal liver [72]. Homozygous knockout embryos exhibit 90% depletion of hematopoietic stem cells (HSCs), suggesting ARID3a is critical for normal numbers of HSC development, and embryos without ARID3a are deficient in erythrocyte development, perhaps explaining lethality [72]. HSCs are included in the hematopoietic stem and progenitor cells (HSPCs), a heterogeneous population of cells that consists of both primitive progenitor cells (HSCs, and multipotent progenitors (MPPs)), and committed progenitor cells (multi-lymphoid progenitors or MLPs and multi-myeloid progenitors or MMPs), and these cells are ultimately responsible for generating all mature hematopoietic cells, including B cells, pDCs, and LDNs (Figure 4). HSPCs from SLE patients have been proposed to be dysfunctional and exhibit defects in proliferation [73,74], as well as deficiencies during hematopoietic transplantation for SLE, which result in the re-emergence of disease and/or engraftment failure [75,76].

Our studies suggest that SLE HSPCs are also associated with the autoimmune dysregulation of ARID3a. ARID3a is expressed at varying frequencies in a subset of cells in all defined progenitor populations of healthy controls [70,71] (Figure 4). In SLE patients, increased numbers of ARID3a^+^ cells existed in all progenitor subpopulations compared to healthy controls, although the total number of circulating hematopoietic progenitors did not differ between SLE patients and healthy individuals [71]. This suggests that ARID3a expression is abnormal, even in early hematopoietic precursors in SLE patients. Other studies found slightly reduced numbers of circulating HSPCs in SLE patients [73,74], but they did not assess ARID3a expression. When HSPCs from SLE patients were transplanted into immunodeficient mice, the engraftment and development were similar between the samples with low numbers of ARID3a-expressing cells and those with high numbers of ARID3a-expressing cells [71], suggesting that ARID3a over-expression does not affect engraftment potential. However, all of the mice that received HSPCs with high numbers of ARID3a-expressing cells generated human ANAs, while only 16% of the mice that received HSPCs with low numbers of ARID3a-expressing cells generated ANAs [71], again associating ARID3a levels with autoantibody production. As mentioned above, SLE patient HSPCs that have increased numbers of ARID3a-expressing cells show increased frequencies of ARID3a expression in all progenitor populations, suggesting that if ARID3a is induced in the early populations of cells, it is either maintained throughout development, or that it is continually induced in maturing subsets.

There are few mechanistic studies defining the differences between healthy and SLE HSPCs. Moonen et al. reported that circulating hematopoietic progenitors, which also have the capacity to repair vascular damage associated with atherosclerosis, exhibit reduced functional capacity in SLE patient-derived samples, but they did not assess hematopoietic development [73]. We found that SLE HSPCs with low numbers of ARID3a^+^ cells proliferated less well compared with both healthy control and SLE samples with increased numbers of ARID3a^+^ HSPCs, possibly because of the lower expression levels of IL7R required for cell proliferation [71]. Importantly, HSPCs can also respond to TLR9 ligands in vitro, with increased expression of both ARID3a and IFNα ([38], unpublished data). IFNα signaling in HSCs induces proliferation and exit from quiescence, and under chronic conditions, reduces the numbers of quiescent HSCs in the bone marrow pool [77,78]. However, others reported that HSCs under chronic IFNα exposure return to quiescence rapidly after initial exposure [79]. Because IFNα can act autonomously on the cells that produce it [80], the associated outcomes of exposure are likely to be diverse.

The importance of ARID3a for B lineage development in mice was demonstrated in multiple systems, and has increased our understanding of ARID3a function [21,24,26,27,28,72]. Several studies revealed that ARID3a expression in B lineage progenitors drives development toward B1 lineage over B2 lineage [21,26]. Interestingly, when ARID3a was overexpressed in murine bone marrow progenitor B cells, the B1a cells that do not require the expression of a surrogate light chain for generation were increased, with those cells showing autoreactive BCR repertoires [21]. Pre-B and immature B cells also exhibited increased expression of *MYC* and *BHLHE41*, and showed decreased expression of *SIGLEC-G* and *CD72* compared with wild type progenitor cells [21]. The reduction of *SIGLEC-G* and *CD72* was hypothesized to contribute to the autoreactive nature of the B1 cells generated, as both are negative regulators of BCR signaling, while the increases in *MYC* and *BHLHE41* expression likely promote the survival of autoreactive B cells [21]. These data provide mechanistic insights for the autoantibody production in ARID3a transgenic mice, and imply that ARID3a expression in B cells from SLE patients may also contribute to the autoantibody phenotype.

ARID3a inhibition in human HSCs also resulted in an increased expression of the myeloid lineage-associated transcription factors *CEBPB* and *CSF1*, as well as a reduction in *TCF3*, the gene for E2A [70]. Furthermore, when ARID3a was overexpressed in cord blood HSPCs, myeloid lineage cell development was impaired [70]. The HSPCs from SLE patients with fewer ARID3a-expressing cells produced fewer B lineage cells in culture compared to both healthy donor cells and SLE HSPCs with high numbers of ARID3a-expressing cells [71]. Conversely, SLE samples with increased numbers of ARID3a-expressing HSPCs showed in vitro expansion equivalent to healthy controls, with increased B lineage cell development [71]. Together, these data suggest that alterations in ARID3a levels during early hematopoiesis in SLE patients could have profound effects on mature cell phenotypes and lineage pathways, as shown in Figure 4, even before known B cell tolerance checkpoints occur.

Elegant studies by a number of laboratories reveal that multiple hematopoietic cell types, which we have not discussed here, are likely to play important roles in SLE pathogenesis, including T cells, macrophages, basophils, and innate lymphoid cells [81,82]. Hematopoiesis is an inter-regulatory process, so these cells may also be affected differentially through interactions with cells that express ARID3a. Indeed, ARID3a expression modifies gene expression pathways in a cell-type specific fashion in the cells in which it is expressed, based on our transcript analyses [38,42,49,70]. To date, we have not observed ARID3a expression in any T cell or monocyte subset; however, our unpublished data show ARID3a transcripts in a subset of NK cells. Our data suggest that ARID3a can contribute to hematopoietic lineage decisions, perhaps resulting in skewing of cell frequencies. Skewing of mature hematopoietic subsets is a common characteristic of SLE [36,50,83,84,85,86], and although we have not definitively shown that ARID3a contributes to that skewing, the data certainly suggest that possibility.

## 6. Regulation of ARID3a Expression

Although ARID3a expression levels clearly change in both healthy inflammatory responses and in multiple SLE cell types, the presumed extracellular triggers that induce ARID3a expression have not been clearly elucidated. Many differentially expressed genes in SLE have been identified through genome-wide associated studies (GWAS), and are associated with single nucleotide polymorphisms (SNPs) that provide a genetic component to the risk of developing the disease [87]. Although SNPs can be found in the coding regions of ARID3a, they do not affect the amino acid sequence, likely because defects in ARID3a function are embryonic lethal in multiple organisms, including mice [72,88,89]. Nearly 80% of the SNPs associated with lupus are located in non-coding DNA [87]. Therefore, it is possible that ARID3a may interact differentially with the regulatory regions affected by these SNPs. This is an area of current interest. A number of studies suggest that epigenetic alterations, including changes in histone acetylation, are associated with SLE [90]. ARID3a has been shown to co-precipitate with histone deacetylases in mice [66], but it is unclear whether it is associated with deacetylases in human SLE cells. Interestingly, in mice, the inhibition of histone deacetylases was effective in reducing autoantibody responses [91]. Clearly, additional studies are required in order to determine how ARID3a regulates other genes and how it is regulated.

Chromatin and other nuclear antigens exposed by NETs are highly opsonized, can activate IFNα responses through TLR9, and have been postulated to trigger autoimmune responses in multiple inflammatory diseases [55,56,92]. Our data indicate that healthy B lineage cells and hematopoietic progenitors respond to TLR9 engagement by increasing ARID3a expression ([38], unpublished data). In early B lineage cells, ARID3a is down-regulated by miRNAs of the 125 family [93], and it is likely to be similarly regulated in pDCs and LDNs, because transcripts for ARID3a are present in healthy control pDCs and LDNs in the absence of detectable ARID3a protein [49]. Let-7 regulates ARID3a levels in mouse hematopoiesis, and controls the switch from fetal to adult B lineage development [21,26], but the role of Let-7 in human B lymphopoiesis is not known. The dysregulation of miRNAs has also been implicated as a contributing factor for disease pathogenesis in SLE [94], and it is possible that ARID3a levels are upregulated in multiple hematopoietic cells in SLE as a consequence of deficiencies in the appropriate regulatory RNAs. Additional studies will be required in order to determine whether ARID3a overexpression in SLE is the result of increased activation, decreased regulation, or a combination of both processes.

## 7. Clinical Implications

The heterogeneous nature of SLE pathogenesis and the lack of understanding of the underlying mechanisms that cause the disease limit therapeutics to those that treat symptoms. Treatments often include glucocorticoids and broad immunosuppressive therapies that act to diminish immune responses as a whole, and perturb normal immune functions against disease. Our data show increased expression of ARID3a in B cells, pDCs, and LDNs from patients with SLE, and associate that increased expression with increases in disease activity. Nothing is known regarding the function or expression of ARID3a in other autoimmune diseases. In a limited patient sample, we did not observe ARID3a over-expression in the B lymphocytes of patients with rheumatoid arthritis [36]. However, ARID3a-expressing cells have been observed in colon cancer [95] and B cell lymphomas [96]. The miRNA Let-7 may regulate ARID3a expression in some cell types, and the dysregulation of Let-7 has been associated with a number of different cancers, where ARID3a expression might also occur [97]. It will be critical to determine in those cases ifARID3a expression is intrinsic to the cancer tissue, or if it is the result of infiltrating immune cells. Many diseases are expected to yield activated immune cells that may express ARID3a in a higher abundance than in healthy tissues.

Our data show strong associations between ARID3a expression and production of IFNα. IFNα is an inflammatory cytokine proposed to play a major role in SLE, and it is currently the target of several clinical trials that indicate that targeting IFNα responses may be beneficial [98,99,100,101]. Our data in B lymphocytes suggest that ARID3a may function upstream of IFNα [38]. Therefore, we propose that new therapies targeting ARID3a directly may be beneficial for the treatment of SLE. Because ARID3a over-expression in SLE is limited to a percentage of cells within any given cell type (i.e., B cells, LDNs, and pDCs), one might imagine that the reversible inhibition of this protein could provide directed therapies that would inhibit the ARID3a-expressing subsets of cells without impairing the normal immune responses in cells that do not express ARID3a. It should be noted that there is no evidence that increases in ARID3a are causally related to SLE disease pathogenesis. However, the plethora of associations of increased ARID3a expression with disease activity in multiple cell types provides strong circumstantial evidence that ARID3a has a major role in SLE pathogenesis, and that ARID3a inhibition may have significant therapeutic benefits in this difficult to treat disease.
